# Efficacy and safety of natural killer cell therapy in patients with solid tumors: a systematic review and meta-analysis

**DOI:** 10.3389/fimmu.2024.1454427

**Published:** 2024-10-16

**Authors:** Heesook Park, Gyurin Kim, Najin Kim, Sungyoen Ha, Hyeonwoo Yim

**Affiliations:** ^1^ Department of Public Health, The Catholic University of Korea, Seoul, Republic of Korea; ^2^ Medical Library, The Catholic University of Korea, Seoul, Republic of Korea; ^3^ Department of Statistics, Sungkyunkwan University of Korea, Seoul, Republic of Korea; ^4^ Department of Preventive Medicine, College of Medicine, The Catholic University of Korea, Seoul, Republic of Korea

**Keywords:** killer cells, natural, NK cells, neoplasms, review, systematic, meta-analysis

## Abstract

**Introduction:**

In 2020, global cancer statistics reported 19.3 million new cases and 10 million deaths annually, highlighting the urgent need for effective treatments. Current therapies, such as surgery, radiation, and chemotherapy, have limitations in comprehensively addressing solid tumor. Recent advances in cancer biology and immuno-oncology, including CAR-T cell therapy, show promise but face efficacy challenges against solid tumors.

**Methods:**

This meta-analysis systematically reviewed studies from PubMed, Embase, Cochrane, and ClinicalTrials.gov databases up to May 2024 to evaluate the clinical efficacy and safety of unmodified NK cell therapies in solid tumors. The included trials focused on reporting objective response rates (ORR).

**Results:**

Thirty-one trials involving 600 patients across various cancers (e.g., NSCLC, HCC, breast, ovarian) were analyzed. NK cell therapies demonstrated promising ORRs, particularly 72.3% in hepatocellular carcinoma, often in combination with local therapies. Safety profiles were favorable, with fatigue being the most common adverse event.

**Discussion:**

NK cell therapies represent a promising treatment option for solid tumors, offering a viable alternative to genetically modified cell therapies like CAR-T. Further research is needed to optimize the clinical utility of NK cell therapy and integrate it effectively into standard cancer treatment regimens.

**Systematic review registration:**

https://www.crd.york.ac.uk/prospero/display_record.php?ID=CRD42023438410, identifier CRD42023438410.

## Introduction

According to the Global Cancer Statistics of 2020, 19.3 million new cases of cancer are diagnosed annually worldwide, with 10 million individuals succumbing to the disease. The most prevalent cancer types include breast cancer (11.7%), lung cancer (11.4%), prostate cancer (7.3%), skin cancer (6.2%), colorectal cancer (6.0%), stomach cancer (5.6%), and liver cancer (4.7%). Notably, lung cancer (18.0%), liver cancer (8.3%), and stomach cancer (7.7%) emerge as the leading causes of cancer-related mortality (1).

Traditional cancer therapies, such as surgery, radiation, and chemotherapy, have been standard treatment options for decades. However, their limitations in addressing the complex nature of cancer are becoming increasingly apparent. Recent advances in cancer molecular biology and immuno-oncology have introduced precision and targeted therapeutics, which aim to selectively eliminate cancer cells while minimizing collateral toxicity and side effects.

One significant advancement is CAR-T cell therapy, where genetically engineered immune cells are designed to recognize and eliminate tumor cells with specific surface antigens. CAR-T therapy has shown remarkable success, especially in managing aggressive B-cell malignancies, offering renewed hope for patients. Nevertheless, this approach faces challenges, including limited efficacy against solid tumors, pronounced toxicities like cytokine release syndrome (CRS), complex manufacturing processes, and high costs (2).

In response, alternative strategies such as CAR-NK and CAR-M cells have been developed (2). These innovative therapies aim to leverage the potential of Natural Killer (NK) cells, key components of the human immune system known for their role in innate immunity through cell-mediated cytotoxicity and antibody-dependent cellular cytotoxicity (3).

Furthermore, NK cells offer several advantages including non-MHC-restricted recognition, the ability to infiltrate tumor microenvironments, potent cytolytic capabilities, and a favorable safety profile with a reduced risk of complications such as CRS, graft-versus- host-disease (GvHD), and immune effector cell-associated neurotoxicity syndrome (ICANS). These attributes make NK cells promising candidates for treating solid tumors (4).

Unlike CAR-T cell therapies, NK cell-based therapies can utilize various sources, including peripheral blood (PB) from healthy donors, umbilical cord blood (UCB), induced pluripotent stem cells (iPSCs), or commercially available NK cell lines. This versatility allows for cost-effective mass production and off-the-shelf availability, providing on-demand treatment options (2, 5).

Given these promising attributes, ongoing research is exploring the potential of NK cells, particularly through the development of CAR-NK cells, marking a new frontier in cancer therapy. This meta-analysis aims to systematically evaluate the clinical efficacy and safety of unmodified NK cells as a treatment modality for solid cancer patients. This meta-analysis represents a critical step toward integrating NK cell-based therapies into clinical practice and facilitating evidence-based decision-making in cancer therapeutics.

## Materials and methods

### Literature search and screening

We conducted a systematic review and meta-analysis to identify published clinical trials that utilized combination therapies involving natural killer cells, as well as trials did not, with a focus on reporting objective response rates. The PubMed, Embase, Cochrane, and ClinicalTrials.gov databases were searched for papers published from database inception through May 2024, using the terms ‘neoplasms’, ‘hematologic neoplasms’ and ‘killer cells, natural’ (**Appendix A**). Additionally, we manually examined references from relevant reviews and articles to avoid omitting any pertinent studies.

The PRISMA guidelines were followed during the review process (6). A prospective protocol was registered in PROSPERO, CRD42023438410.

### Inclusion and exclusion criteria

The selection criteria included: (1) articles reporting prospective clinical trials which were published before the literature review date; (2) participants were diagnosed solid cancer; (3) articles in which participants were treated with natural killer cells; and (4) clinical trials reporting the objective response rate. Studies not matching the selection criteria were excluded. Exclusion criteria were: (1) studies without full-text available; (2) non-interventional studies; (3) studies lacking efficacy assessment parameters; and (4) studies in which participants were treated with agents other than NK cells. HS Park and KR Kim independently conducted the literature search and data extraction. Discrepancies were resolved through discussion with a senior author (HW Yim). For publications reporting duplicate populations, we only included the most recent study or the study reporting the most complete efficacy data.

### Data extraction

Data extraction was conducted using standardized, pre-piloted forms for study tabulation and quality assessment. Independent investigators (HS Park and KR Kim) extracted the following information: study characteristics (first author, year of publication, if applicable, country, phase, study design, patient number, age, gender, cancer type, previous treatment history, cell origin, expansion duration, infusion dose, dose schedule, adverse events, and treatment response (complete response, partial response, stable disease, progressive disease, objective response rate [ORR], disease control rate [DCR]), and survival (progression-free survival [PFS], overall survival [OS], 6-month and 1-year survival rates). ORR was defined as the sum of all patients demonstrating complete or partial response divided by the total of evaluable patients, and DCR was defined as the sum of all patients demonstrating complete or partial response or stable disease divided by the total of evaluable patients.

### Outcome measurement

The primary outcome was objective response rate (ORR), defined as the sum of all patients demonstrating complete or partial response divided by the total of evaluable patients. The secondary outcome was disease control rate (DCR), defined as the sum of all patients demonstrating complete or partial response or stable disease divided by the total of evaluable patients. Safety endpoint was adverse events of any grade, as reported by the individual trials.

### Subgroup analysis

Several subgroup analyses were conducted to examine the impact of various factors on the efficacy of NK cell immunotherapy in solid cancer. The subgroups included cancer type, age, study year, subject number, cell origin, expansion duration, infusion dose, IL-2 administration, lymphodepleting chemotherapy regimen, combination treatment. Subgroups were analyzed if they appeared in at least two studies.

### Statistical analysis

We performed a meta-analysis using Comprehensive Meta-analysis 4.0. Event rate (ER) with 95% confidence intervals (CIs) were calculated for each outcome. Pooled ORR and DCR were calculated using fixed- and random-effects models depending on the heterogeneity across the included studies (7). The heterogeneity was assessed using I2 values. Generally, I2 values of 25% represent low heterogeneity, and I2 values of 50% and 75% are evidence of moderate and high heterogeneity, respectively. When no statistically significant heterogeneity existed, the analysis was calculated with a fixed-effect model; otherwise, a random-effect model was used. P values of <0.05 were considered statistically significant (8). We performed subgroup analysis to assess the efficacy of natural killer cells with different clinical parameters in all available studies.

The analysis of safety data, the secondary evaluative variable, was conducted using R software version 4.3.1 (R Foundation for Statistical Computing, Vienna, Austria). The “epiR” package was employed to calculate the incidence rate and its corresponding 95% confidence interval.

Begg’s and Egger’s tests were used to assess publication bias and a publication bias was indicated by p-value *<* 0.05 in Begg’s or Egger’s values (9, 10). Sensitivity analyses were conducted using both fixed and random effects models.

The quality of the included studies was evaluated using the ROBINS-I tool (Risk of Bias in Non-Randomized Studies of Interventions), which assesses the risk of bias across seven domains: confounding, participant selection, intervention classification, deviations from intended interventions, missing data, outcome measurement, and selection of reported results. Each study was categorized as having low, moderate, or high risk of bias. Risk of bias was independently assessed by two reviewers, with disagreements resolved by a third reviewer. To explore the impact of study bias on outcomes, we conducted a separate analysis of studies classified as having low or moderate risk of bias (11).

## Results

### Selection of clinical trials

A total of 3076 publications were retrieved through the initial literature search, and 1905 studies remained after duplications were excluded. After reviewing titles and abstracts, 1774 publications were excluded because the topics were irrelevant, the articles were reviews, the studies were not used NK cells as an intervention, or no usable data were reported. 131 potentially relevant articles were identified for detailed review.

After a full-text review, 100 studies were excluded for the following reasons: 52 lacked full text or relevant information, 37 involved patients with pre-existing partial response (PR) or higher responsiveness, 8 were used cells other than NK cells, 3 were non-intervention studies.

**Figure 1:**
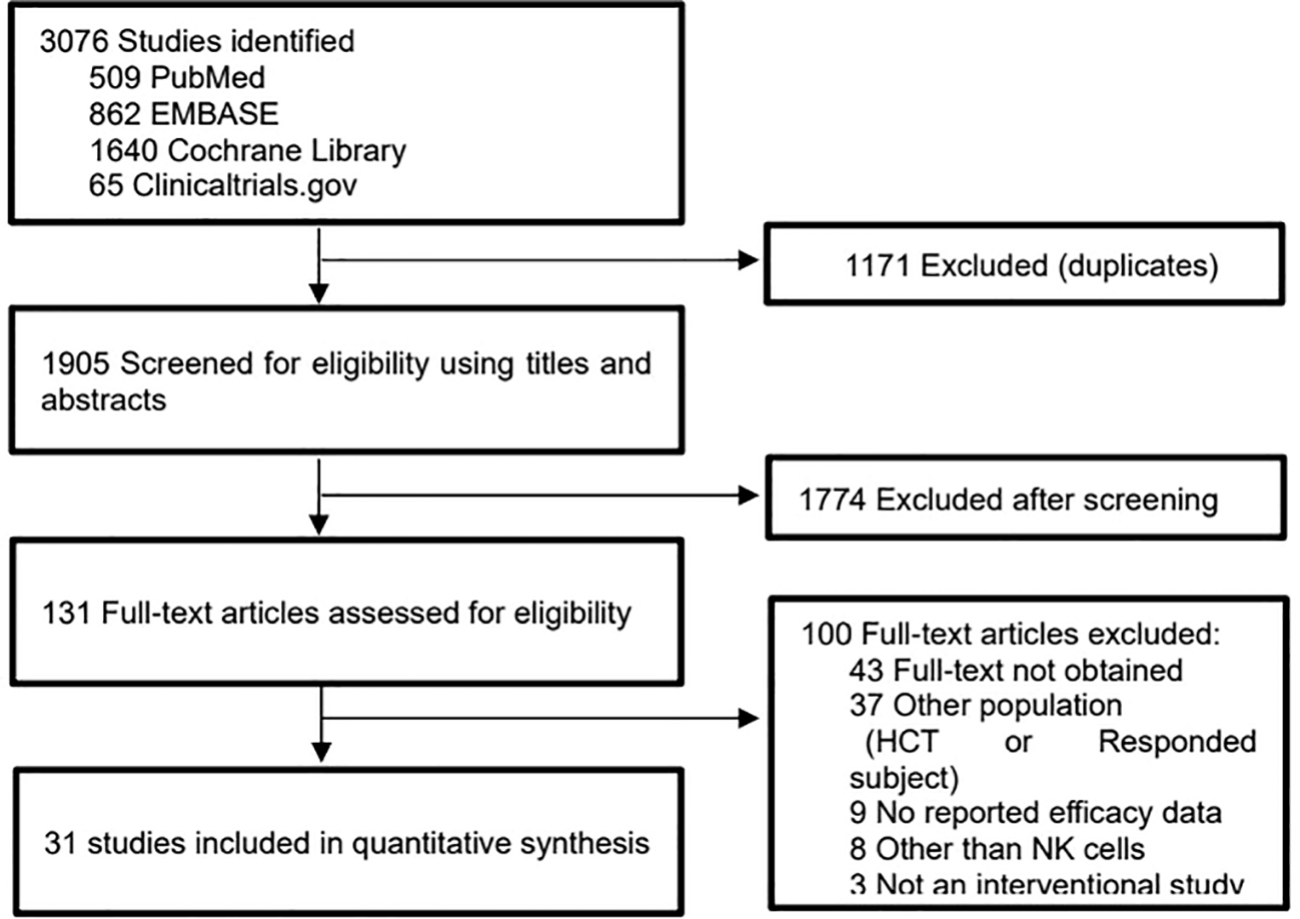
Flow diagram representing the selection process of studies.

After this process, 31 clinical trials involving 600 patients were identified as eligible to be included in the meta-analysis (**Figure 1**) (12–42).

### Characteristics of the included trials

A total of 600 patients were included in this meta-analysis from 31 selected references. All the studies focused on solid tumor patients, and they were further classified by cancer type, with 7 studies involving Non-Small Cell Lung Cancer (NSCLC), 3 studies on Hepatocellular Carcinoma (HCC), and 2 studies each on Breast and Ovarian Cancer, Pancreatic Cancer, Renal Cell Carcinoma (RCC), Central Nervous System Tumors (CNS tumor), and Digestive System Cancers. One study was centered on Nasopharyngeal Cancer, while 9 studies encompassed all solid tumor types. Regarding the origin of cells, 15 studies used allogeneic cells, 12 used autologous cells, 2 employed cell lines, and 1 study utilized both allogeneic and autologous cells. Additionally, 8 studies incorporated lymphodepleting chemotherapy prior to NK cell infusion, 8 combined NK cell therapy with IL-2, and 19 studies combined NK cell therapy with other anticancer treatments. These characteristics reflect the diversity within the meta-analysis, covering patient demographics, cell sources, cancer types, and the incorporation of various treatment approaches.

All included 31 clinical trials reported the outcomes of NK cell treatment. 15 trials with 218 patients reported the number of therapeutic toxicities of NK cell. The characteristics of all included studies are summarized in **Table 1**.

**Table 1:** Basic characteristics of included clinical trials.

Study ID (Year)	Patient number (^*^)	Age (years)	Gender M/(F)	Type of Cancer	Origin	Duration of expansion	Cytokines for expansion	Dose	Lymphodepleting chemotherapy	IL-2 administration	Combination therapy	Clinical outcome	Ref.
Jia, L. 2022	20	49-71	17 (3)	NSCLC	Auto	14 days	IL-2, IL-15	3×10^9 cells every 3 weeks	No	No	Sintilimab	CR1 PR8 SD5 PD6	(21)
Bae, W. K. 2022	11	43-71	10 (1)	HCC	Auto	14 days	IL-2, IL-15	2.5×10^8, 5×10^8, 10×10^8 cells for 5 consecutive days	Cy + Flu	No	5-FU + Cis	CR4 PR3 SD2 PD2	(14)
Otegbeye, F. 2022	7	23-66	2 (5)	CRC (6) Sarcoma (1)	Allo	14~21days	IL-2	1X10^7/kg, 2.5X10^7/kg, 5X10^7/kg	Cy + Flu	No	No	SD2 PD5	(36)
Lim, C. M. 2022	7	45-66	6 (1)	Nasopharyngea ca.	Auto	10 days	IL-2	1 × 10^6 cells/kg → 1 × 10^7 cells/kg 3 times a week for 2 weeks	No	Yes 1MU/m2	Cetuximab	SD4 PD3	(28)
Kim, E. J. 2022	14 (12)	49-73	11 (3)	NSCLC	Auto	17~18 days	–	2×10^9 or 4×10^9 cells every 1 week	No	No	Pembrolizumab	PR5 SD3 PD4	(23)
Nagai, K. 2020	10	22-61	4 (6)	Mixed solid cancer	Auto	14 days	IL-2	10^6 cells → 10^7 cells → 10^8 cells every 2 weeks	No	No	No	PR1 SD3 PD6	(35)
Lee, S. C. 2020	31	32-73	–	Mixed advanced HER2-positive solid tumors refractory to standard therapy (breast ca. (30), gastric ca. (1))	Allo	–	–	1X10^6cells/kg,1X10^7 cells/kg, 5X10^7 cells/kg, 1X10^8 cells/kg X 3~19 infusions	No	Yes 1 million IU/m2	Trastuzumab with or without Bevacizumab	PR1 SD16 PD14	(24)
Khatua, S. 2020	9	8-18	6 (3)	CNS ca. Recurrent pediatric medulloblastoma and ependymoma	Auto	–	–	1 × 10^6/m2, 1 × 10^7/m2, X 3 infusions weekly 3 × 10^3/m2 X 1 infusion weekly	No	No	No	SD2 PD7	(22)
Lin, M. 2020	55	56.0-69.0	34 (21)	NSCLC	Allo	12 days	IL-2	–	No	No	Pembrolizumab	PR20 SD30 PD5	(32)
Yang, Y. 2019	18	57^**^	11 (7)	HCC	Allo	8~12 days	No	–	No	No	IRE	CR3 PR13 SD2	(41)
Ishikawa, T. 2018	9 (6)	34-79	3 (32)	Digestive ca. gastric ca. (3), colorectal ca. (6)	Auto	18~24 days	IL-2	0.5 × 10^9, 1.0 × 10^9, 2.0 × 10^9 cells every 3 weeks	No	No	IgG1 antibody, Capecitabine or S-1, Cisplatin, Trastuzumab, Capecitabine or S-1, Oxaliplatin, Cetuximab	SD4 PD2	(20)
Liang, S. 2018	27	around 55 yrs old	5 (8)	NSCLC	Allo	12 days	No	–	No	No	Cetuximab	PR4 SD17 PD6	(25)
Alnaggar, M. 2018	20	31-77	13 (31)	HCC	Allo	12 days	No	8~10X10^9 cells D 13, 14, 15	No	No	IRE	CR5 PR8 SD5 PD2	(12)
Liang, S. 2017	32	26-71	0 (18)	Breast ca.	Allo	12 days	No	–	No	No	Cryoablation with or without Herceptin.	CR3 PR12 SD10 PD7	(26)
Federico, S. M. 2017	13	15.96-75.46	5 (8)	CNS ca. Recurrent/Refractory Neuroblastoma	Allo	–	No	4.7 X 10^6/kg ~ 59.5 X 10^6/kg D 7 or 8 every 2 cycles	No	Yes No further detail	Cyclophosphamide/Topotecan, Irinotecan/Temozolomide, Ifosfamide/Carboplatin/Etoposide, Hu14.18K322A	CR3 PR5 SD5	(15)
Lin, M. 2017	37	57^**^	36 (31)	Pancreatic ca.	Allo	12 days	No	8~10X10^9 cells D 13, 14, 15 for 2 cycles	No	No	IRE	CR5 PR12 SD10 PD10	(29)
Lin, M. 2017	30	47^**^	12 (18)	NSCLC	Allo	12 days	No	8~10X10^9 cells D 13, 14, 15 for 2 cycles	No	No	Mixed Cryoablation	CR7 PR12 SD6 PD5	(31)
Lin, M. 2017	20	57^**^	12 (8)	Pancreatic ca.	Allo	12 days	No	8~10X10^9 cells D 13, 14, 15 for 2 cycles	No	No	IRE	CR6 PR10 SD4	(30)
Lin, M. 2017	30	61^**^	18 (12)	RCC	Allo	12 days	No	8~10X10^9 cells D 13, 14, 15 for 2 cycles	No	No	Cryoablation	CR7 PR17 SD5 PD1	(33)
Liang, S. 2017	36	around 50 yrs old	0 (36)	Breast ca.	Mixed	12 days	No	–	No	No	No	PR4 SD22 PD10	(27)
Yang, Y. 2016	20 (15)	48-78	16 (4)	Mixed advanced, recurrent solid tumors	Allo	14 days	IL-2	1 X 10^6 cells/kg, 1X10^7 cells/kg single infusion, 1X10^6 cells/kg, 3X10^6 cells/kg, 1X10^7 cells/kg, 3X10^7 cells/kg X 1 infusion/week for 3 weeks	No	No	No	SD7 PD8	(40)
Sakamoto, N. 2015	14 (12)	48-78	11 (3)	Digestive ca. un-resectable, locally advanced and/or metastatic digestive cancer	Auto	21~22 days	IL-2	0.5 × 10^9, 1.0 × 10^9, 2.0 × 10^9 cells D 0, 7, 14	No	No	None or S-1	SD5 PD7	(38)
Tonn, T. 2013	15	9-71	6 (9)	Mixed predominantly end-stage solid tumors	Cell line NK-92 cells	~12 days	IL-2	1X10^9, 3X10^9, 1X10^10 cells/m2 2 infusion (2h, 50h)	No	No	No	SD1 PD14	(39)
Yang, Y. J. 2013	19	45-75	12 (7)	NSCLC	Auto	14 days	IL-2	2.0×10^9 cells D 1, 8 every 3 weeks	No	No	Docetaxel	PR2 SD12 PD5	(42)
Parkhurst, M. R. 2011	8	21-56	4 (4)	Mixed metastatic melanoma (7), renal cell carcinoma (1)	Auto	21 days	IL-2	4.7X10^10 (± 2.1X10^10) cells	Cy + Flu	Yes 720,000 IU/kg	No	CR0 PR0 SD- PD-	(37)
Geller, M. A. 2011	20	30-65	0 (20)	Mixed refractory metastatic breast ca. (6), ovarian ca. (14)	Allo	14 days	No	2.16X10^7 cells/kg (8.33X10^6 – 3.94X10^7)	Cy + Flu	Yes 10 MU	No	PR4 SD12 PD3	(16)
Iliopoulou, E. G. 2010	16 (15)	50-75	11 (5)	NSCLC	Allo	21~23 days	IL-15	0.2 ~ 29 X 10^6/kg	No	No	Cisplatinum, pPaclitaxel, Docetaxel, Gemcitabine, Pemetrexed, Vinorelbine.	PR2 SD6 PD7	(18)
Arai, S. 2008	12	31-74	8 (4)	Mixed Renal cell cancer (11), malignant melanoma (n=1)	Cell line NK-92 cells	15~17 days	IL-2	1X10^8/m2, 3X10^8/m2, 1X10^9/m2 3X10^9/m2 D 1, 3, 5	No	No	No	SD4 PD8	(13)
Miller, J. S. 2005	43 (23)	–	–	Mixed metastatic melanoma (10), metastatic renal cell carcinoma (13), refractory Hodgkin (1), poor-prognosis AML (19)	Allo	–	IL-2	–	Cy + Flu	Yes 1.75 X 10^6 IU/m2	No	CR0 PR0 SD6 PD-	(34)
Ishikawa, E. 2004	9	23~70	5 (4)	CNS ca. malignant glioma; anaplastic gliomas (6), glioblastoma (3)	Auto	14 days	IL-2	injected into tumor cavities (0.4~2.3 x 10^9 cells) combined with intravenous injection (0.2~3.7 x 10^9 cells) weekly	No	Yes less than 100 IU/kg	No	PR2 SD3 PD4	(19)
Hercend, T. 1990	12	25-68	11 (1)	RCC	Auto	28~35 days	IL-2	7~125 X 10^9 every 3 weeks	No	Yes 3 X 10^6 U/m2	No	PR3 SD3 PD6	(17)

^*^ no. of subjects for tumor response evaluation, ^**^median.

### ORR and DCR of NK cell treatment outcome

**Figure 2:**
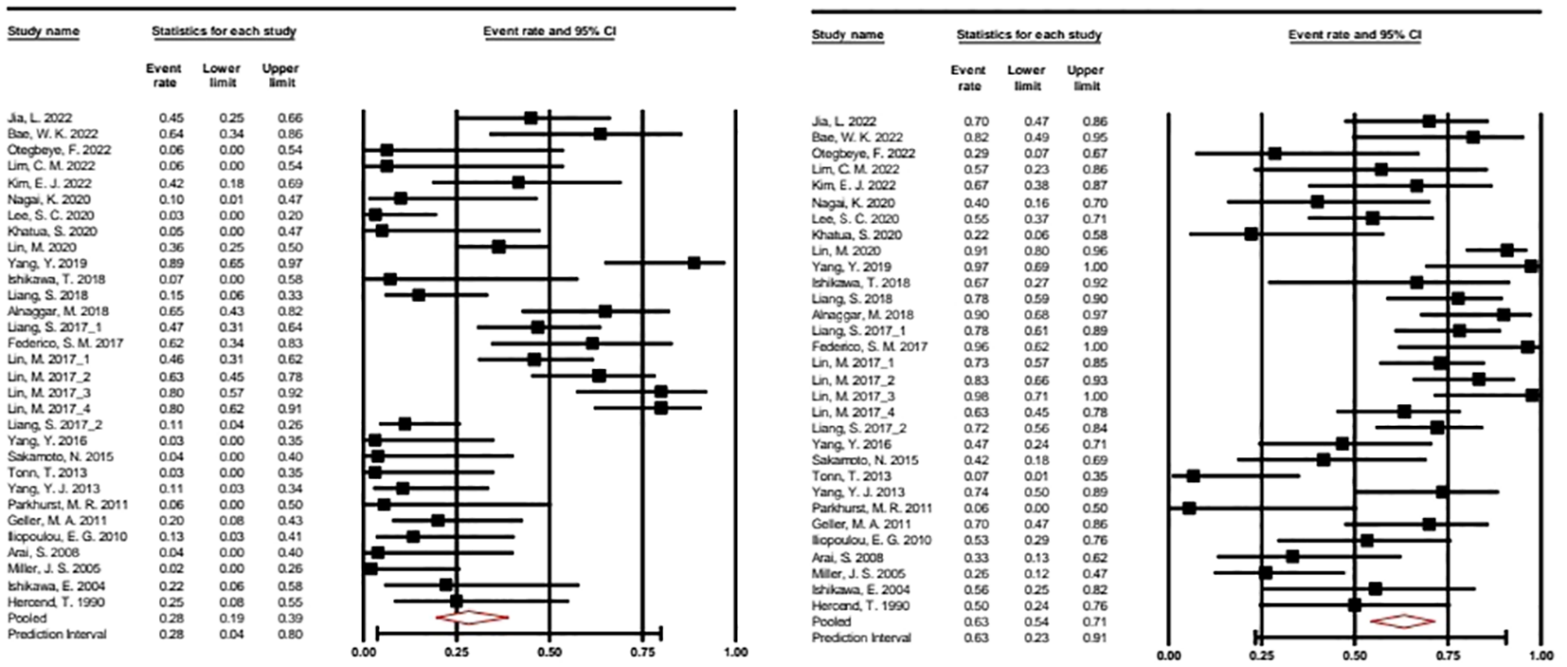
Forest plot of the overall response rate and disease control rate with 95% CI. A random effects model was applied. ORR: 0.282 (95% CI: 0.914–0.389). Heterogeneity: I2 = 77.192%; p = 0.000. DCR: 0.632 (95% CI: 0.543–0.713). Heterogeneity: I2 68.347%; p = 0.000. CRR, Complete response rate; ORR, Overall response rate.

Forest plots showing the best ORR and DCR with 95% CI are presented in **Figure 2**.

In our meta-analysis of 31 selected studies, we examined both Overall Response Rate (ORR) and Disease Control Rate (DCR), using the event rate as the effect size index. To account for potential study variability, we applied a random-effects model, considering these studies as a random sample from a broader universe of similar research. For ORR, the mean effect size was calculated at 0.282 (95% CI: 0.194 - 0.389), while for DCR, it is determined as 0.632 (95% CI: 0.543 - 0.713). These values represent the response rates across the studies. Heterogeneity analysis indicated significant variability among the included studies for both ORR (I2 = 77%) and DCR (I2 = 68%), with Q-tests for heterogeneity producing highly significant results (p < 0.001) in both cases.

### Subgroup analysis based on various clinical factors

Due to a considerable heterogeneity detected in ORR and DCR, we conducted several comprehensive subgroup analyses and metaregressions according to the protocol designed at the beginning of the study. The results of subgroup analyses and metaregressions based on different factors are presented in **Table 2**, including the number of studies, the number of patients, pooled ORR and DCR with 95% CI, heterogeneity and p-value for metaregression. The random effects model was applied due to significant heterogeneity as shown in **Table 2**.

**Table 2:** Subgroup analyses and metaregressions based on different factors.

Clinical factors	Studies number	Patients number	Pooled ES (95% CI)	Heterogeneity		Metaregression
				I2 static	P-value (Q statistic)	p-value
**ORR**	31	591	0.282 (0.194 - 0.389)	77.192	0.000	
**DCR**	31	591	0.632 (0.543 - 0.713)	65.347	0.000	
Study year-ORR						
>2000	21	453	0.384(0.268-0.515)	78.895	0.000	0.289(0.204-0.391) Q-value 9.580, df 1 (p=0.002), I2 78.246
≤2010	5	66	0.107(0.046-0.227)	0.000	0.478	
Subject number-ORR						
>20	9	301	0.319(0.177-0.505)	85.093	0.000	0.251(0.175-0.347) Q-value 7.519, df 2 (p=0.023), I2 77.192
≤20	15	234	0.341(0.198-0.520)	76.646	0.000	
≤10	7	56	0.103(0.045-0.219)	0.000	0.906	
Age-ORR						
≥12	28	546	0.291(0.199-0.403)	77.557	0.000	0.291(0.200-0.402) Q-value 0.003, df 1 (p=0.960), I2 76.932
<12	2	22	0.274(0.014-0.911)	79.154	0.029	
Type of cancer-ORR						
HCC	3	49	0.723(0.527-0.859)	36.038	0.209	0.357(0.266-0.459) Q-value 18.898, df 2 (p=0.000), I2 77.192
NSCLC	7	178	0.318(0.190-0.481)	72.165	0.001	
Others	21	364	0.188(0.103-0.319)	77.769	0.000	
Origin-ORR						
Allogeneic	16	393	0.396(0.261-0.549)	81.494	0.000	0.248(0.178-0.333) Q-value 11.860, df 2 (p=0.003), I2 77.192
Autologous	12	135	0.217(0.121-0.359)	51.050	0.021	
Others	3	63	0.087(0.037-0.333)	0.000	0.551	
Expansion duration-ORR						
>2wks	7	77	0.164(0.077-0.317)	30.799	0.193	0.306(0.217-0.412) Q-value 4.791, df 1 (p=0.029), I2 76.692
≤2wks	19	431	0.381(0.262-0.516)	79.938	0.000	
Dose-ORR						
≥10^9	11	223	0.458(0.305-0.620)	74.873	0.000	
<10^9	6	74	0.157(0.049-0.406)	65.262	0.013	0.353(0.239-0.486) Q-value 5.807, df 2 (p=0.055), I2 73.610
Mixed	5	50	0.167(0.038-0.502)	66.858	0.017	
LD-ORR						
Yes	5	69	0.157(0.037-0.470)	70.557	0.009	0.285(0.197-0.395) Q-value 1.023, df 1 (p=0.312), I2 77.192
No	26	522	0.303(0.206-0.421)	78.226	0.000	
IL-2-ORR						
Yes	8	123	0.160(0.066-0.342)	63.483	0.008	0.285(0.198-0.391) Q-value 2.665, df 1 (p=0.103), I2 77.192
No	23	468	0.330(0.223-0.459)	78.634	0.000	
Comb. Therapy-ORR						
Local Tx.	6	155	0.699(0.555-0.813)	64.576	0.015	0.300(0.236-0.373) Q-value 49.575, df 2 (p=0.000), I2 77.295
Systemic Tx.	12	248	0.295(0.190-0.428)	66.434	0.001	
No	12	176	0.124(0.078-0.190)	0.000	0.594	
Risk of bias-ORR						
Low	19	417	0.420(0.301-0.550)	78.224	0.000	0.276(0.200-0.368) Q-value 16.718, df 1 (p=0.000), I2 77.192
Moderate	12	174	0.103(0.053-0.190)	31.797	0.136	
Study year-DCR						
>2010	21	453	0.384(0.268-0.515)	78.895	0.000	0.289(0.204-0.391) Q-value 9.580, df 1 (p=0.002), I2 78.246
≤2010	5	66	0.107(0.046-0.227)	0.000	0.473	
Subject number- DCR						
>20	9	301	0.709(0.581-0.810)	73.309	0.000	0.616(0.531-0.693) Q-value 8.353 df 2 (p=0.015), I2 68.347
≤20	15	234	0.657(0.517-0.774)	65.235	0.000	
≤10	7	56	0.413(0.265-0.578)	20.191	0.276	
Age- DCR						
≥12	28	546	0.652(0.568-0.727)	63.081	0.000	
<12	2	22	0.704(0.027-0.995)	86.868	0.006	0.652(0.569-0.727) Q-value 0.011 df 1 (p=0.917), I2 64.748
Type of cancer- DCR						
HCC	3	49	0.889(0.749-0.956)	0.000	0.426	0.668(0.595-0.734) Q-value 17.102 df 2 (p=0.000), I2 68.347
NSCLC	7	178	0.760(0.653-0.843)	49.108	0.067	
Others	21	364	0.533(0.427-0.637)	64.467	0.000	
Origin- DCR						
Allogeneic	16	393	0.719(0.603-0.812)	73.861	0.000	0.628(0.544-0.705) Q-value 5.133 df 2 (p=0.077), I2 68.347
Autologous	12	135	0.561(0.442-0.674)	36.253	0.101	
Others	3	63	0.355(0.079-0.780)	85.461	0.001	
Expansion duration- DCR						
>2wks	7	77	0.484(0.353-0.616)	18.284	0.290	0.644(0.567-0.714) Q-value 8.916 df 1 (p=0.003), I2 62.713
≤2wks	19	431	0.723(0.636-0.796)	60.503	0.000	
Dose- DCR						
<10^9	6	223	0.542(0.342-0.729)	54.352	0.052	0.599(0.497-0.693) Q-value 1.699 df 2 (p=0.428), I2 61.269
≥10^9	11	74	0.671(0.522-0.792)	67.245	0.001	
Mixed	5	50	0.539(0.358-0.709)	32.907	0.202	
LD- DCR						
Yes	5	69	0.438(0.182-0.731)	75.594	0.003	0.642(0.556-0.720) Q-value 1.868 df 1 (p=0.172), I2 68.347
No	26	522	0.659(0.571-0.738)	65.055	0.000	
IL-2- DCR						
Yes	8	123	0.522(0.353-0.686)	59.490	0.016	0.628(0.541-0.708) Q-value 2.090 df 1 (p=0.148), I2 68.347
No	23	468	0.665(0.566-0.751)	67.872	0.000	
Comb. Therapy- DCR						
Local Tx.	6	155	0.819(0.681-0.906)	57.220	0.039	0.664(0.590-0.730) Q-value 19.352 df 2 (p=0.000), I2 68.526
Systemic Tx.	12	248	0.729(0.636-0.806)	46.149	0.040	
No	12	176	0.406(0.276-0.550)	62.997	0.002	
Risk of bias-DCR						
Low	19	417	0.737(0.652-0.807)	57.423	0.001	0.631(0.555-0.700) Q-value 15.081 df 1 (p=0.000), I2 68.347
Moderate	12	174	0.440(0.323-0.565)	51.561	0.019	

Sub-analysis for ORR showed statistically significant results, with a 72.3% (0.527-0.859) ORR in hepatocellular carcinoma patients and 69.9% (0.555-0.813) in patients receiving combination with local treatment, and allogenic NK cells showed better ORR at 39.6% (0.261-0.549) than autologous NK cells. In addition, 38.1% (0.262-0.516) showed a better response rate when the culture period was within 2 weeks, and 45.8% (0.305-0.620) when the number of cells administered was ≥10^9. Contrary to expectations, the use of lymphodepletion or IL-2 administration demonstrated significantly lower ORR, with values of 30.3% (95% CI: 0.206-0.421) and 33.0% (95% CI: 0.223-0.459), respectively.

These results showed a similar trend in the sub-analysis of DCR.

### Overall incidence of AEs

**Figure 3:**
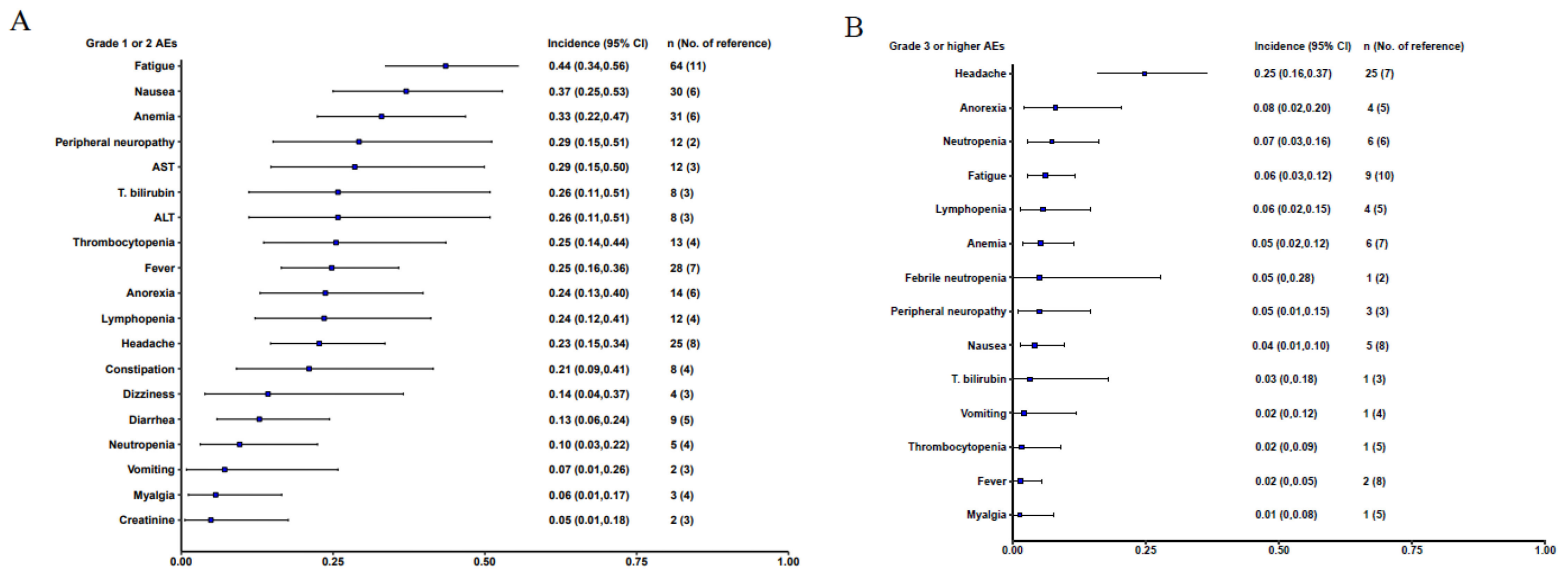
Forest plot of the overall incidence AEs with 95% CI. **(A)**. Grade 1 or 2 adverse events; **(B)** Grade 3 or higher adverse event.

The meta-analysis included 15 out of the 31 studies, comprising a total of 218 participants, where adverse events with specified Grades were reported. Among Grade 1-2 adverse events, fatigue was the most common, at 44% (95% CI: 0.34-0.56), followed by nausea at 37% (95% CI: 0.25-0.53) and anemia at 33% (95% CI: 0.22-0.47). Grade 3 or higher adverse reactions included Headache occurred in 25% (95% CI: 0.16-0.37), Anorexia in 8% (95% CI: 0.02-0.20), and neutropenia at 7% (95% CI: 0.03-0.16). Notably, no cases of Cytokine Release Syndrome (CRS) or Graft-versus-Host Disease (GvHD), common side effects of cell therapies like CAR-T, were reported (**Figure 3**).

### Publication bias, sensitivity analysis, and risk of bias

Begg’s rank correlation and Egger’s regression analyses were performed to evaluate publication bias. A publication bias was indicated by p-value < 0.05 in Begg’s or Egger’s tests. Publication bias was detected for ORR. In the sensitivity analysis, a significant difference was observed in the pooled ES for the best ORR and DCR based on the fixed and random effects models (**Table 3**).

**Table 3:** Publication bias and sensitivity analysis.

Outcome indicators	Publication bias		Pooled effect size (95% CI)	
	p-value (Begg’s test)	p-value (Egger’s test)	Random effect model	Fixed effect model
ORR	0.33321	0.00038	0.282(0.194-0.389)	0.398(0.350-0.449)
DCR	0.19302	0.30573	0.632(0.543-0.713)	0.646(0.600-0.689)

**Figure 4:**
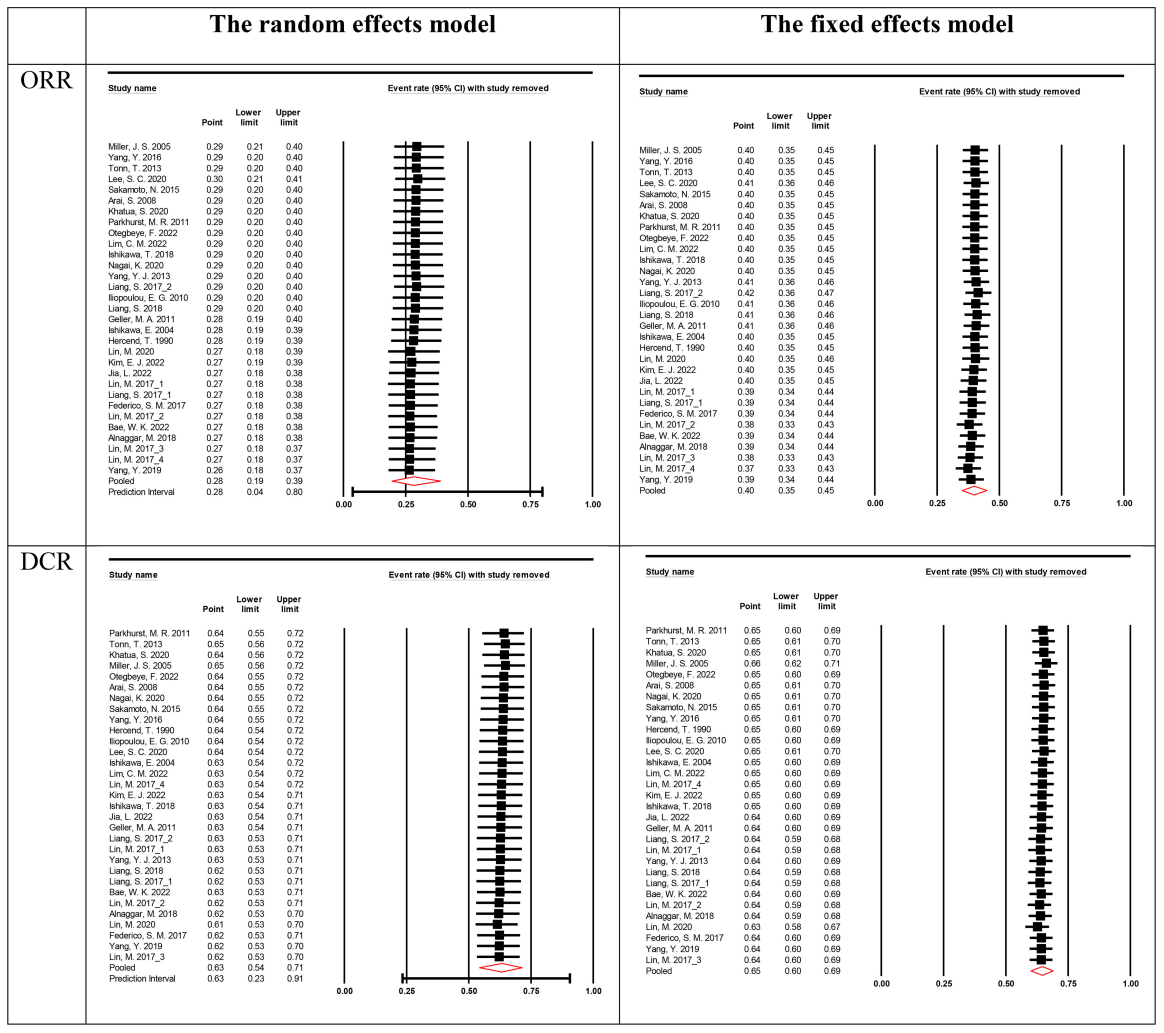
Sensitivity analysis for the best overall response rate. The vertical line on the left indicated the total lower CI, the vertical line in the middle indicated the total pooled effect size and the vertical line on the right indicated the total higher CI. The circle indicated the pooled effect size after deleting the study.

Sensitivity analyses were performed to assess the stability of the results by sequentially removing each study. The removal of any single study did not change the overall statistical results, indicating that the results of this study were statistically robust (**Figure 4**).

Using the ROBINS-I tool, we assessed the risk of bias in 19 studies as low and in 12 studies as moderate (**Appendix B**). This thorough evaluation enabled us to explore the relationship between study quality and reported outcomes, specifically Overall Response Rate (ORR) and Disease Control Rate (DCR). Our analysis revealed a clear pattern: studies with low risk of bias consistently demonstrated more favorable outcomes (**Tables 2**, **3**).

## Discussion

In our study, we conducted a meta-analysis and systematic review of 31 studies involving the administration of non-genetically modified natural killer (NK) cells to patients with solid tumors. The objective response rate (ORR), defined as a response of partial response or better, was 28.2% (95% CI: 0.194 - 0.389), and the disease control rate (DCR), defined as stable disease or better, was 63.2% (95% CI: 0.543 - 0.713).

The meta-analysis included studies ranging from the early stages of NK cell research starting in 1999 to advancements in technology up to 2020, without limiting the timeframe. Consequently, the more recent studies from 2011 to 2020 showcased an ORR of 38.4% (95% CI: 0.268-0.515) and a DCR of 72.7% (95% CI: 0.648-0.794), suggesting the possibility that advancements in NK cell manufacturing technology and related developments may contribute to the enhanced efficacy of cell therapy, indicating the potential of NK cells as a new option for solid tumor patients without effective treatments.

Particularly noteworthy were the high ORR of 72.3% (95% CI: 0.527-0.859) and DCR of 88.9% (95% CI: 0.749-0.956) observed in hepatocellular carcinoma. Among the three clinical trials involving liver cancer patients, two included irreversible electroporation (IRE) and one involved a combination with intrahepatic arterial chemotherapy. When NK cells were combined with local therapies, such as IRE, the observed ORR was 69.9% (95% CI: 0.555-0.813), and the DCR was 81.9% (95% CI: 0.681-0.906). Notably, these outcomes exhibited superior efficacy compared to the concomitant use of systemic treatments (ORR 0.295, 95% CI: 0.190-0.428; DCR 0.729, 95% CI: 0.636-0.806) or administration of NK cells alone (ORR 0.124, 95% CI: 0.078-0.190; DCR 0.406, 95% CI: 0.276-0.550). This underscores the heightened effectiveness achieved through the strategic selection of combination therapies, emphasizing their potential in maximizing the therapeutic impact of NK cells. Hence, choosing appropriate combination therapies tailored to each cancer type is crucial to optimizing the therapeutic efficacy of NK cells.

Cytokines such as IL-2 and IL-15 play a role in the development of NK cell cytotoxic function and stimulation of NK cell proliferation when administered during cell culture or in combination with cells (43–46). Among the 31 studies included in the meta-analysis, 16 studies used IL-2, two studies used both IL-2 and IL-15, and one study used IL-15 for NK cell expansion. Additionally, eight studies administered IL-2 to patients. Despite IL-2 being recognized for its critical role in immune system activation and induction of tumor cell death through Fc γ receptor binding, our meta-analysis revealed lower ORR and DCR when combined with IL-2, with respective values of 0.160 (95% CI: 0.066-0.342) and 0.522 (95% CI: 0.353-0.686), compared to non-combined scenarios (ORR 0.330, 95% CI: 0.223-0.459; DCR 0.665, 95% CI: 0.566-0.751) (47).

One of the known function of IL-2 is to promote T cell proliferation. However, at low doses, IL-2 selectively stimulates the expansion of regulatory T cells (Tregs). This increase in Tregs can suppress the overall immune response against tumors by inhibiting the activity of cytotoxic T cells and NK cells. Although the exact doses of IL-2 used in the studies were not specified, it is plausible that low-dose IL-2 contributed to reduced NK cell efficacy by promoting Treg expansion. Elevated Treg levels may have suppressed NK cell activity, thereby diminishing the effectiveness of NK cell therapy (48).

Similar trends were observed regarding the use of lymphodepleting chemotherapy (LD). Although LD is commonly used in CAR-T cell therapy to enhance therapeutic efficacy, it was performed in five clinical cases in our meta-analysis (49). Surprisingly, our meta-analysis found that cases without LD showed higher ORR 30.3% (95% CI: 0.206-0.421) and DCR 66.5% (95% CI: 0.566-0.751) compared to those with LD (ORR 15.7%, 95% CI: 0.037-0.470; DCR 43.8%, 95% CI: 0.182-0.731) (50).

Despite potential biases due to limited sample sizes and study numbers, the observed results raise questions about the application of IL-2 and LD in solid tumors, indicating the need for further investigation.

Regarding NK cell sourcing, allogeneic cells demonstrated superior efficacy compared to autologous cells (ORR 39.6%, 95% CI: 0.261-0.549; DCR 71.9%, 95% CI: 0.603-0.812 vs. ORR 21.7%, 95% CI: 0.121-0.359; DCR 56.1%, 95% CI: 0.442-0.674). This enhanced efficacy of allogeneic NK cells can be attributed to the donor-recipient incompatibility in killer cell immunoglobulin-like receptors (KIRs) and major histocompatibility complex (MHC) class I. In allogeneic settings, the mismatch between the KIRs on donor NK cells and the MHC class I molecules on recipient cells disrupts the inhibitory signaling that would normally suppress NK cell activity. This mismatch leads to greater NK cell activation, allowing the allogeneic NK cells to target and destroy tumor cells more effectively. Furthermore, autologous NK cells are often derived from patients who have received extensive prior cancer treatments, like chemotherapy or radiation. These treatments can impair NK cell function by causing exhaustion, altering their phenotype, and reducing their cytotoxic potential. As a result, NK cells from pre-treated patients may be less effective compared to those from healthy donors, further limiting the efficacy of autologous NK cell therapy (51, 52).

The advantages of allogeneic NK cells, particularly their off-the-shelf potential, were highlighted, considering the lengthy and costly production process of CAR-T cells. Although recent developments aim to simplify the manufacturing process and explore allogeneic CAR-T cells, challenges such as graft-versus-host disease (GvHD) persist. Notably, NK cell characteristics demonstrated higher efficacy without reports of GvHD or cytokine release syndrome (CRS) (53).

The most commonly reported adverse event was fatigue, highlighting the safety advantages of NK cells. Through this meta-analysis, we have demonstrated that non-genetically modified NK cells can be an effective and safe option for solid tumors. The diverse results from subgroup analyses provide insights into various considerations for the future development of NK cell therapies.

Our Robins-I analysis revealed a consistent trend of improved ORR and DCR in studies with low risk of bias, emphasizing the importance of reducing bias for reliable outcomes. Both ORR and DCR analyses revealed a significant improvement in studies with low bias, highlighting a stronger treatment effect. This emphasizes the importance of reducing bias for reliable outcomes and underscores the need for rigorous study design and execution when evaluating treatment efficacy.

## Strengths and weaknesses

### Strengths

Pioneering Exploration in Solid Tumor Patients: This study represents the first meta-analysis to explore the efficacy of NK cells specifically in solid tumor patients. While there have been meta-analyses on NK cell administration in hematologic cancers, this study breaks new ground by focusing on solid tumor patients. Considering the expanding scope of cell therapies, including NK cells, beyond hematologic malignancies, this research is poised to contribute significantly.

Conducting Diverse Subgroup Analyses: To address potential heterogeneity from studies spanning an extended period, this research conducted diverse subgroup analyses. By exploring factors such as study timelines, cell origins, cell culture durations, and cancer types, the study aimed to identify sources of heterogeneity and investigate efficacy outcomes based on various characteristics.

Robustness in Pre-defined Sensitivity Analyses: The key findings demonstrated robustness through pre-defined sensitivity analyses.

No Restriction on Publication Years: By including all research on NK cell therapy up to the present without restricting publication years, the study provides a comprehensive overview of the field.

### Weaknesses

Inherent Challenges in Cross-Trial Comparisons: Ensuring comparability across clinical trials conducted over several decades is challenging. Given the diverse solid tumor types included and changes in standard therapies for these cancers over the 20-year period, the study may not be directly applicable to contemporary clinical decision-making.

Confirmed Heterogeneity and Potential Bias in Analyses: Despite using random-effect models to account for heterogeneity, the diversity in concomitant treatment and patient populations may introduce potential biases. Despite conducting various subgroup analyses, differences in study designs might still impact the results.

## Conclusion

This meta-analysis confirms the efficacy of NK cell administration in patients with solid tumors, demonstrating a significant increase in Overall Response Rate (ORR) and Disease Control Rate (DCR). Additionally, the safety profile of NK cell therapy is reinforced by the absence of significant adverse events, such as Graft-versus-Host Disease (GvHD) and Cytokine Release Syndrome (CRS).

It is crucial to consider variations in efficacy based on cancer types and combination therapies for informed treatment decisions. Looking ahead, cell-based therapies, particularly those involving advanced genetic manipulation of NK cells, represent a pivotal frontier in drug development. Refining NK cells, especially through the use of allogeneic cells, promises not only enhanced efficacy but also a favorable toxicity profile. This progress is expected to lead to the development of optimized NK cells as off-the-shelf products, ushering in a transformative new era in cell-based therapies. Continued exploration and integration of these advancements are essential for improving patient outcomes and revolutionizing therapeutic strategies.

## Data Availability

The original contributions presented in the study are included in the article/supplementary material. Further inquiries can be directed to the corresponding author.
