# Supplementary material for: Efficacy and safety of natural killer cell therapy in patients with solid tumors: a systematic review and meta-analysis
Source: Front Immunol. 2024 Oct 16;15:1454427. doi: 10.3389/fimmu.2024.1454427 (PMC11522797; doi:10.3389/fimmu.2024.1454427)
Supplement: Supplementary file 2 [file Table2.docx]

| **Study ID (Year)** | **Risk of bias by Domain** | | | | | | **Overall risk of bias** | **Concerns** | **Ref.** |
| --- | --- | --- | --- | --- | --- | --- | --- | --- | --- |
|  | **Confounding** | **Selection participants** | **Classifying exposure** | **Deviation from intended intervention** | **Missing data** | **Measuring outcomes** |  |  |  |
| Jia, L. 2022 | low | low | low | low | low | low | low |  | [21] |
| Bae, W. K. 2022 | low | low | Low | low | low | low | low |  | [14] |
| Otegbeye, F. 2022 | moderate | low | low | low | low | low | moderate | Cancer type | [36] |
| Lim, C. M. 2022 | low | low | low | low | low | low | low |  | [28] |
| Kim, E. J. 2022 | low | low | low | low | moderate | low | moderate | ITT analysis | [23] |
| Nagai, K. 2020 | moderate | low | low | low | low | low | moderate | Cancer type | [35] |
| Lee, S. C. 2020 | moderate | low | low | low | low | low | moderate | Number of IP administration and combination therapy | [24] |
| Khatua, S. 2020 | low | low | low | low | low | low | low |  | [22] |
| Lin, M. 2020 | low | low | low | low | low | low | low |  | [32] |
| Yang, Y. 2019 | low | low | low | low | low | low | low |  | [41] |
| Ishikawa, T. 2018 | moderate | low | low | low | moderate | low | moderate | Cancer type and combination therapy,  ITT analysis | [20] |
| Liang, S. 2018 | low | low | low | low | low | low | low |  | [25] |
| Alnaggar, M. 2018 | low | low | low | low | low | low | low |  | [12] |
| Liang, S. 2017 | low | low | low | low | low | low | low |  | [26] |
| Federico, S. M. 2017 | low | low | low | low | low | low | low |  | [15] |
| Lin, M. 2017 | low | low | low | low | low | low | low |  | [29] |
| Lin, M. 2017 | low | low | low | low | low | low | low |  | [31] |
| Lin, M. 2017 | low | low | low | low | low | low | low |  | [30] |
| Lin, M. 2017 | low | low | low | low | low | low | low |  | [33] |
| Liang, S. 2017 | low | low | low | low | low | low | low |  | [27] |
| Yang, Y. 2016 | moderate | low | low | low | low | low | moderate | Cancer type | [40] |
| Sakamoto, N. 2015 | low | low | low | low | low | low | low |  | [38] |
| Tonn, T. 2013 | moderate | low | low | low | low | low | moderate | Cancer type | [39] |
| Yang, Y. J. 2013 | low | low | low | low | low | low | low |  | [42] |
| Parkhurst, M. R. 2011 | moderate | low | low | low | low | low | moderate | Cancer type | [37] |
| Geller, M. A. 2011 | moderate | low | low | low | moderate | low | moderate | Cancer type,  ITT analysis | [16] |
| Iliopoulou, E. G. 2010 | Moderate | low | low | low | low | low | moderate | Combination therapy | [18] |
| Arai, S. 2008 | moderate | low | low | low | low | low | moderate | Cancer type | [13] |
| Miller, J. S. 2005 | moderate | low | low | low | low | low | moderate | Cancer type | [34] |
| Ishikawa, E. 2004 | low | low | low | low | low | low | low |  | [19] |
| Hercend, T. 1990 | low | low | low | low | low | low | low |  | [17] |
